# The validity and responsiveness of the ICECAP-A capability-well-being measure in women with irritative lower urinary tract symptoms

**DOI:** 10.1007/s11136-015-1225-y

**Published:** 2016-01-11

**Authors:** Ilias Goranitis, Joanna Coast, Hareth Al-Janabi, Pallavi Latthe, Tracy E. Roberts

**Affiliations:** Health Economics Unit, Institute of Applied Health Research, Public Health Building, University of Birmingham, Birmingham, B15 2TT UK; School of Social and Community Medicine, University of Bristol, Bristol, UK; School of Clinical and Experimental Medicine, University of Birmingham, Birmingham, UK; Birmingham Women’s NHS Foundation Trust, Birmingham, UK

**Keywords:** ICECAP-A, EQ-5D-3L, Psychometric validation, Outcome valuation, Economic evaluation, Overactive bladder

## Abstract

**Purpose:**

A desire to incorporate broader aspects of well-being in health economic evaluations has led to the development of the ICEpop CAPability measure for Adults (ICECAP-A). The ICECAP-A draws upon Amartya Sen’s capability approach and conceptualises well-being as the capability to achieve *Stability, Attachment, Autonomy, Achievement,* and *Enjoyment*. The aim of this study was to assess the psychometric performance of the ICECAP-A in a context where patient outcomes can extend beyond health-related quality of life.

**Methods:**

Longitudinal data were collected for 478 women with symptoms of urinary frequency and urgency, with or without incontinence. Women were recruited across 22 hospitals in the UK and had a mean age of 55 (SD 14). The psychometric performance of the measure was evaluated in relation to the EuroQol Five-Dimension Questionnaire (EQ-5D-3L) and the International Consultation on Incontinence Questionnaire for Overactive Bladder (ICIQ-OAB) and involved an assessment of acceptability, construct validity, and responsiveness using parametric and nonparametric methods.

**Results:**

ICECAP-A showed good convergence with the ICIQ-OAB with 20 out of 22 expected patterns of relationship confirmed. Findings suggested that the ICECAP-A has better discriminative properties than EQ-5D-3L and as good as those of the ICIQ-OAB, confirming expected associations with clinical and demographic factors. The ICECAP-A was more responsive than EQ-5D-3L and ICIQ-OAB to deteriorations of clinical symptoms. Improvements in symptoms were not valued as highly as deteriorations by either ICECAP-A or EQ-5D-3L.

**Conclusions:**

The ICECAP-A is a valid and responsive measure capturing broad emotional and practical impacts of urinary symptoms on women’s well-being and could be considered for use in economic evaluations in this context.

## Introduction

Consideration of health-related quality of life (HrQoL) is an integral component of healthcare decision-making in many systems of the developed world. HrQoL, however, may offer limited scope when interventions result in wider personal well-being gains [1–4] or in external effects on groups other than the patient [5, 6]. One appropriate framework for conceptualising these broader well-being impacts for health policy purposes is the capability approach [7, 8]. The capability approach was developed by Amartya Sen as a basis for assessing well-being in terms of what people do and are (functioning) and particularly, what people are able to do and be (capability) in their lives [9]. While a number of capability measures have been developed [10–14], the ICEpop^1^ CAPability (ICECAP) measures are distinct as they provide a generic measure of capability-well-being for use in the economic evaluation of health and social care interventions.

The ICECAP measure for the general adult population (ICECAP-A) has recently been developed [12] and conceptualises well-being as the capability of an individual to achieve the valuable functionings of *Stability, Attachment, Autonomy, Achievement,* and *Enjoyment,* with health potentially being a direct determinant of functioning. Previous validation work on the ICECAP-A has suggested that the attributes of the measure can comprehensively capture quality of life [15] and that the measure is able to identify expected differences in capability-well-being in a general population sample [16]. In terms of responsiveness, small changes in capability-well-being were evident as a result of changes in physical and psychological health after a knee pain intervention [17].

However, no evidence for the psychometric properties of the ICECAP-A exists in a clinical context where there are likely to be impacts on well-being more broadly than those captured by conventional HrQoL measures. This paper explores the acceptability, construct validity, and responsiveness of the ICECAP-A in relation to the three-level EuroQol Five-Dimension Questionnaire (EQ-5D-3L) [18] and the International Consultation on Incontinence Questionnaire for Overactive Bladder (ICIQ-OAB) [19] in women with irritative lower urinary tract symptoms (LUTS) involving urinary frequency and urgency, with or without incontinence. The impact of these symptoms on HrQoL is well established [20, 21], but broader well-being issues may arise as a result of shame, embarrassment, discomfort, and lack of confidence [22]. It is, therefore, possible that such effects may be missed by HrQoL measures, but picked up by measures of broader capability-well-being.

## Methods

### Data source

The paper relied on data from the largest observational study undertaken to estimate the accuracy and cost-effectiveness of bladder ultrasound scan in the diagnosis of detrusor overactivity [23]. Detrusor overactivity is termed the involuntary contraction of the detrusor muscle observed during the filling phase of urodynamic studies and is perceived to be one of the main causes of LUTS. The study was carried out in 22 hospitals across the UK, and women were recruited if they presented increased frequency of urination and mild to severe urgency, with or without urinary incontinence. Exclusion criteria involved pregnancy or up to 6 weeks post-partum, stress-predominant mixed incontinence, continuous medical treatment, like antimuscarinics, for more than 6 months, and a surgical treatment or urodynamic studies during the past 6 months for a bladder condition. Women in the study had a transvaginal bladder ultrasound scan (index test) followed by urodynamic studies (reference test). Women were initially treated conservatively. All women provided written informed consent and were followed up for a year.

### Outcome measures

The outcome measures used in the analysis included the ICECAP-A, EQ-5D-3L, and ICIQ-OAB. These measures were administered prior to diagnostic testing at baseline and 6-month follow-up, while the latter two were additionally administered at the 12-month follow-up. More information about the different measures is provided below.

#### ICEpop CAPability measure for adults (ICECAP-A)

The ICECAP-A is a generic and preference-based measure of capability-well-being [12]. It comprises five conceptual attributes (*Stability, Attachment, Autonomy, Achievement,* and *Enjoyment*) with each having four response options that range from full capability to no capability. Individual responses to the five attributes can subsequently be translated into a capability index score using a UK population value set obtained using the best–worst scaling method [24]. The capability index scores range from 0 to 1, indicating no capability and full capability, respectively.

#### EuroQol Five-Dimension Questionnaire (EQ-5D-3L)

The EQ-5D-3L is a generic and preference-based measure of HrQoL [18], comprising five conceptual attributes (*Mobility, Self*-*care, Usual activities, Pain and discomfort,* and *Anxiety and depression*). Each attribute has three response options ranging from no problems to severe problems. Responses to the EQ-5D-3L are used to derive a health index score based on country-specific value sets, which represent general population preferences for the different health states. In this study, health index scores were calculated using the UK value set obtained based on the time trade-off method [25]. The scores range from −0.594 to 1, depending on whether severe problems or no problems are reported across the five dimensions of the instrument. On this scale, the values of 0 and 1 represent death and full health, respectively, while values lower than 0 represent health states considered to be worse than death.

#### International Consultation on Incontinence Questionnaire for Overactive Bladder (ICIQ-OAB)

The ICIQ-OAB is a urinary incontinence-specific measure of quality of life [19]. This measure asks four questions, each having five response options. The questions relate to: (a) the frequency of urination during the day, (b) frequency of nocturia, (c) frequency of having to rush to the toilet for urination, and (d) frequency of leaking before getting to the toilet. Responses to these questions are scored from 0 to 4, whereby a higher score reflects increased frequency (severity) of symptoms. A total ICIQ-OAB score is derived by adding the scores from all responses and thus can range from 0 to 16. Each of the four questions has a second part intended to measure, on an 11 (0–10)-point Likert scale, the level of ‘bother’ from the different symptoms. Although responses to these questions are not included in the scoring of the instrument, they are helpful in determining patient’s priority for treatment or monitoring changes over time.

### Psychometric analysis

The sample size was determined by the main study [23], which aimed to recruit at least 600 women after loss to follow-up. The psychometric properties of the ICECAP-A were assessed in relation to the EQ-5D-3L and ICIQ-OAB and involved explorations of acceptability, construct validity, and responsiveness. Analyses for this research were based upon women who responded at both baseline and 6-month follow-up, allowing for the same sample to be used in all analyses. No data imputation was performed, and all analyses were carried out in Stata version 12MP.

#### Acceptability

Acceptability is a term used to reflect the perceived relevance of an outcome measure to the respondents in certain clinical contexts. Generic outcome measures, such as the ICECAP-A and EQ-5D-3L, are developed for application in all clinical contexts, and, therefore, demonstrating high levels of acceptability is an important quality. The acceptability of the ICECAP-A was approximated through the completion rates at baseline and 6-month follow-up [26], with rates above 95 % indicating high levels of acceptability [27].

#### Validity

Construct validity relates to the degree that relationships between a measure and other factors confirm a priori expected patterns of relationship and comprises both convergent and discriminative (known group) validity [28]. Convergent validity assesses the extent of correlation between instruments intended to measure similar or overlapping constructs [28]. The convergence between the ICECAP-A, EQ-5D-3L, and ICIQ-OAB index scores was explored using Pearson’s correlation coefficients. Spearman rank correlation coefficients were used for the convergence across dimension scores and between index and dimension scores. Correlations were considered strong if the coefficient was above 0.5, moderate if the coefficient was between 0.3 and 0.5, and weak if the coefficient was below 0.3 [29]. Given that the EQ-5D-3L attributes are scored from no problems (lowest level) to severe problems (highest level), and the ICECAP-A attributes from no capability (lowest level) to full capability (highest level), the scoring of the EQ-5D-3L dimensions was reversed for the purposes of this analysis in order to allow for a more intuitive interpretation of findings.

Discriminative or known-group validity assesses the extent to which instruments are able to distinguish between dissimilar constructs [28], namely constructs differing in a trait likely to be associated with women’s quality of life. The constructs used in the analysis related to age, body mass index (BMI), presence of detrusor overactivity, previous urinary surgery, and presence of prolapse or voiding dysfunction. The four questions included in the ICIQ-OAB, which indicate how bothersome the frequencies of the different urinary symptoms are to women, and which are not considered as part of the scoring process of the ICIQ-OAB, were also used to construct known groups. To test whether the mean index scores of the three measures differed between known groups, a univariate analysis using one-way ANOVA and a Kruskal–Wallis *H* test was undertaken. To account for potential confounding problems associated with univariate analyses, a multivariate regression analysis was additionally carried out using age, BMI, past surgery, presence of detrusor overactivity, advance prolapse, and voiding dysfunction as covariates.

#### Responsiveness

Given that a fundamental principle underpinning healthcare interventions is the improvement of health and well-being, it is important that instruments are also valid in a longitudinal context. In the assessment of responsiveness, the different measures are compared for patient groups expected to have experienced a change in health and well-being based on an external criterion (anchor) [26]. Three analyses were undertaken to explore the responsiveness of the ICECAP-A using different anchors of potential clinical change.

In the first analysis, changes in the scores of the three outcome measures were assessed based on changes in the mean self-reported ‘bother’ across individual urinary symptoms in the ICIQ-OAB [30]. In this analysis, responsiveness was assessed for the overall sample and for specific subgroups (those with the same, decreased and increased level of ‘bother’). In the second analysis, changes in the scores of the ICECAP-A and EQ-5D-3L were assessed relative to changes in the actual ICIQ-OAB score and thus based on changes in the frequency of urinary symptoms. This analysis explored changes in capability and health index scores for those of whom ICIQ-OAB score decreased (symptoms less frequent), increased (symptoms more frequent), and remained the same. In the third analysis, changes in the scores of the three measures were assessed based on whether women felt that symptoms were ‘improved’, ‘deteriorated’, or ‘without change’ on a retrospective transition question.

In the absence of a gold-standard measure of HrQoL and well-being, responsiveness was evaluated using the standardised response mean (SRM) effect size statistic, calculated as the ratio of the mean change between baseline and follow-up index scores to the standard deviation of the change scores [26, 31]. Alternative methods for assessing responsiveness, such as the receiver operating characteristic (ROC) curve analysis, which require a gold-standard anchor, were not explored, as none of the anchors of this study can be considered an appropriate reference standard of a valued change of clinical symptoms by the general public, which is inherent in the valuation of preference-based outcome measures. Paired *t* tests and Wilcoxon rank sum tests were also carried out to identify significant changes in scores. The values 0.2, 0.5, and 0.8 were used as thresholds for small, moderate, and large SRM statistics [32]. Floor and ceiling effects were calculated as the proportion of women selecting the response options indicating the lowest (floor effect) or highest (ceiling effect) level of quality of life across all attributes of each questionnaire.

### Hypothetical constructs

Good measurement validation practices require an a priori statement of hypotheses on the expected relationship between the theoretical concepts explored [33, 34]. Therefore, hypothetical constructs were developed independently by each author in the light of available evidence and personal judgment before seeing any of the results. These are available in ‘Appendices 1 and 2’. The two overarching expectations were that the ICECAP-A would show better convergence with the condition-specific measure than the EQ-5D-3L and that the ICECAP-A would be more sensitive in identifying differences and changes in the level of ‘bother’ from urinary symptoms.

## Results

The primary study recruited 687 women with lower urinary tract symptoms. Responses to at least one of the outcome measures were provided by 655 (95.3 %) women at baseline and 478 (69.6 %) at the 6-month follow-up period. The results presented in this section are based on women who responded to at least one of the outcome measures at both baseline and 6-month follow-up (n = 478). Women had a mean age of 55 (SD 14) and a mean weight of approximately 77 kg (SD 18), with 198 (41.4 %) women being classified as obese based on their BMI. Most women (44.8 %) were diagnosed with detrusor overactivity, had no evidence of prolapse (74.2 %), and no voiding difficulties (56.4 %). A significant proportion of women (73.2 %) reported high levels of ‘bother’ from urinary symptoms and had no previous urinary surgery (82.4 %). More information about the sample characteristics is provided in Table 1.

**Table 1:** Sample characteristics (N = 478)

Variable	Category	Frequency (%)
Age (mean 54.69; SD 13.89)		
Age groups	<30	27 (5.65)
	30–44	90 (18.83)
	45–64	236 (49.37)
	≥65	125 (26.15)
Weight (mean 76.83; SD 17.87)		
BMI	Normal	115 (24.06)
	Overweight	165 (34.52)
	Obese	198 (41.42)
Urodynamic diagnosis	Detrusor overactivity	208 (44.73)
	Mixed incontinence	84 (18.06)
	Stress incontinence	56 (12.04)
	Normal bladder	76 (16.34)
	Other^a^	41 (8.82)
Advanced prolapse	No	351 (74.21)
	Yes	122 (25.79)
Voiding difficulty	No	265 (56.38)
	Yes	205 (43.62)
Previous urinary surgery	No	389 (82.42)
	Yes	83 (17.58)
Impact of symptoms (‘bother’)	Low	42 (8.99)
	Moderate	83 (17.77)
	High	342 (73.23)

^**a**^Includes the diagnoses of voiding dysfunction and low compliance

### Acceptability

Missing data for the ICECAP-A attributes ranged between 1.3 % *(Autonomy)* and 1.9 % *(Enjoyment)* at baseline, and between 3.8 % *(Achievement)* and 4.6 % *(Attachment)* at 6-month follow-up. For the EQ-5D-3L, missing data ranged between 0.6 % *(Mobility* and *Self*-*care)* and 0.8 % *(Pain and discomfort* and *Anxiety and depression)* at baseline, and between 3.3 % *(Self*-*care* and *Anxiety and depression)* and 4 % *(Pain and discomfort)* at 6-month follow-up. For the ICIQ-OAB, 0–1.9 % of values was missing at baseline and 0–1.3 % at 6-month follow-up. In all instances, completion rates were greater than 95 % indicating a high level of acceptability.

### Construct validity

The convergence between the three outcome measures is given in Table 2. A strong correlation was found between the capability and health index scores, and all attributes of the EQ-5D-3L were found to have a moderate to strong correlation with the ICECAP-A index score. All correlations between the ICECAP-A and EQ-5D-3L were statistically significant at the 1 % level, apart from correlations between the ICECAP-A attribute of *Attachment* and the EQ-5D-3L attributes of *Mobility, Usual activities,* and *Pain and discomfort*. For the latter two, however, correlations were statistically significant at the 5 % level.

**Table 2:** Convergent validity between the ICECAP-A, EQ-5D-3L, and ICIQ-OAB (n = 478)

	*ICECAP*-*A*						*ICIQ*-*OAB*				
	Capability index score	Stability	Attachment	Autonomy	Achievement	Enjoyment	ICIQ-OAB score	Frequency of urination (day)	Frequency of urination (night)	Frequency of rush	Frequency of leak
EQ-5D											
Health index score	0.53**	0.38**	0.21**	0.48**	0.45**	0.40**	−0.26**	−0.06	−0.20**	−0.11*	−0.15**
Mobility	0.35**	0.23**	0.06	0.41**	0.36**	0.29**	−0.21**	−0.05	−0.18**	−0.03	−0.11*
Self-care	0.35**	0.20**	0.13**	0.44**	0.32**	0.28**	−0.21**	−0.04	−0.14**	−0.10*	−0.20**
Usual activities	0.38**	0.26**	0.11*	0.40**	0.31**	0.34**	−0.25**	−0.14**	−0.11*	−0.16**	−0.21**
Pain and discomfort	0.30**	0.21**	0.10*	0.27**	0.28**	0.22**	−0.17**	−0.02	−0.18**	−0.11*	−0.12**
Anxiety and depression	0.50**	0.46**	0.30**	0.30**	0.40**	0.42**	−0.09*	−0.05	−0.11*	−0.02	−0.06
ICIQ-OAB											
ICIQ-OAB score	−0.28**	−0.23**	−0.12*	−0.19**	−0.21**	−0.25**					
Frequency of urination (day)	−0.10*	−*0.06*	−0.08	−*0.06*	−0.08*	−0.16**					
Frequency of urination (night)	−0.15**	−0.11*	−0.03	−0.12*	−0.13**	−0.15**					
Frequency of rush	−0.13**	−0.12**	−0.04	−0.10*	−0.12**	−0.11*					
Frequency of leaking	−0.17**	−0.18**	−0.09*	−0.13**	−0.14**	−0.13**					

A priori hypothesised correlations between ICECAP-A and ICIQ-OAB are shown in italics

Correlations >0.5 are considered strong; ≤0.5 and ≥0.3 moderate; <0.3 weak [29]

* Correlations significant at the 5 % level

** Correlations significant at the 1 % level

Correlations between the ICECAP-A index score and ICIQ-OAB, although being slightly higher than those between the EQ-5D-3L index score and ICIQ-OAB (apart from the case of frequency of nocturia), were of similar strength. From the 17 hypothesised associations between the ICECAP-A attributes and ICIQ-OAB (Appendix 1), only the correlations between the frequency of urination during the day and the attributes of *Stability* and *Autonomy* were not statistically significant. In addition to the hypothesised correlations, other significant correlations were found. *Attachment* was significantly correlated at the 5 % level with the ICIQ-OAB score and the frequency of leaking before urination. Finally, frequency of nocturia was found to have a significant correlation with *Autonomy* (5 % level of significance), *Achievement* and *Enjoyment* (1 % level of significance). All correlations were in the expected direction (Appendix 1).

The results on the discriminative validity of the different outcome measures are presented in Table 3. According to the a priori hypotheses (Appendix 2), the ICECAP-A was expected to be able to discriminate among the categories of BMI, detrusor overactivity, and the different variables related to self-reported levels of ‘bother’ from urinary symptoms. There were significant differences in terms of both ICECAP-A and EQ-5D-3L among the categories of BMI. The presence of detrusor overactivity was significantly associated with lower levels of capability-well-being (at the 5 % level), but only in the univariate analysis. Significantly lower levels of HrQoL (at the 1 % level) were also evident for those with detrusor overactivity. Statistically significant differences in capability-well-being were evident between those with high and low levels of ‘bother’ from the different urinary symptoms, apart from the symptom of urgency. These differences were also captured by the ICIQ-OAB, but not from the EQ-5D-3L, which only identified significant differences in HrQoL (at the 5 % level) for the urinary frequency symptom, and only in the univariate analysis.

**Table 3:** Discriminative (known group) validity of the ICECAP-A, EQ-5D-3L, and ICIQ-OAB (n = 478)

Variables	Categories	ICECAP-A			EQ-5D			ICIQ-OAB		
		Mean (SD)	*P* value^§^	*P* value^§§^	Mean (SD)	*P* value^§^	*P* value^§§^	Mean (SD)	*P* value^§^	*P* value^§§^
Age	<65	0.82 (0.18)	0.12	0.14	0.69 (0.28)	0.18	0.17	9.14 (2.72)	0.53	0.43
	≥65	0.85 (0.15)			0.65 (0.28)			9.31 (2.55)		
BMI	Normal	0.85 (0.15)	0.00** ^*,†*^	0.01*	0.75 (0.24)	0.00**^,††^	0.00**	8.86 (2.56)	0.06^†^	0.09
	Overweight	0.85 (0.15)			0.69 (0.28)			8.99 (2.63)		
	Obese	0.80 (0.19)			0.64 (0.30)			9.53 (2.75)		
Detrusor overactivity	No	0.85 (0.16)	0.02* ^*,†*^	*0.07*	0.72 (0.26)	0.00**^,††^	0.00**	8.73 (2.54)	0.00**^,††^	0.00**
	Yes	0.81 (0.18)			0.63 (0.31)			9.76 (2.73)		
Surgery	No	0.83 (0.17)	0.59	0.37	0.69 (0.28)	0.35	0.65	9.13 (2.64)	0.29	0.05
	Yes	0.84 (0.17)			0.66 (0.28)			9.47 (2.85)		
Advance prolapse	No	0.83 (0.17)	0.97	0.81	0.69 (0.29)	0.16	0.18	9.32 (2.64)	0.06^†^	0.02*
	Yes	0.83 (0.18)			0.65 (0.30)			8.79 (2.70)		
Voiding difficulty	No	0.83 (0.16)	0.92	0.96	0.69 (0.27)	0.55	0.69	9.06 (2.69)	0.28	0.13
	Yes	0.83 (0.18)			0.68 (0.30)			9.33 (2.63)		
Bother—frequency of urination (day)	≤5	0.87 (0.13)	0.00** ^*,††*^	0.01*	0.74 (0.20)	0.03*	0.07	6.86 (2.16)	0.00**^,††^	0.00**
	>5	0.82 (0.19)			0.67 (0.30)			9.77 (2.47)		
Bother—frequency of urination (night)	≤5	0.87 (0.13)	0.00** ^*,†*^	0.02*	0.73 (0.23)	0.02*	0.10	7.44 (2.21)	0.00**^,††^	0.00**
	>5	0.81 (0.18)			0.67 (0.30)			9.85 (2.53)		
Bother—frequency of rush	≤5	0.87 (0.14)	*0.06* ^*†*^	0.14	0.73 (0.22)	0.19	0.29	7.00 (2.37)	0.00**^,††^	0.00**
	>5	0.82 (0.17)			0.68 (0.29)			9.52 (2.56)		
Bother—frequency of leaking	≤5	0.88 (0.13)	0.00** ^*,††*^	0.04*	0.74 (0.25)	0.10	0.31	7.20 (2.36)	0.00**^,††^	0.00**
	>5	0.82 (0.18)			0.68 (0.29)			9.54 (2.57)		
Total impact of symptoms	Low	0.86 (0.14)	0.00** ^*,††*^	0.01**	0.75 (0.19)	0.15	0.27	6.31 (2.17)	0.00**^,††^	0.00**
	Moderate	0.87 (0.13)			0.70 (0.24)			7.57 (2.15)		
	High	0.81 (0.18)			0.67 (0.30)			9.94 (2.39)		

A priori hypothesised significant differences between known groups in the ICECAP-A index score are shown in italics

^**§**^Results of univariate analysis

^**§§**^Results of multivariate regression analysis using age, BMI, detrusor overactivity, surgery, advance prolapse, and voiding difficulty as covariates

* Significant differences between groups at the 5 % level using one-way ANOVA

** Significant differences between groups at the 1 % level using one-way ANOVA

^†^Significant differences between groups at the 5 % level using a Kruskal–Wallis *H* test

^††^Significant differences between groups at the 1 % level using a Kruskal–Wallis *H* test

### Responsiveness

The responsiveness of the three measures for all women and by self-reported change in the level of ‘bother’ is given in Table 4. There were no floor effects evident for the three measures. There was some evidence of ceiling effect for the EQ-5D-3L, with 16 % of women at baseline and 21 % at 6-month follow-up reporting full health. Approximately 12 % of women reported full capability at the two time periods. Across the three responsiveness analyses, the ICECAP-A appeared to be more responsive than the EQ-5D-3L, but with effect sizes being trivial to small. More specifically, for women with the same and, particularly, increased level of ‘bother’, the ICECAP-A was found to be more responsive in comparison with the EQ-5D-3L and ICIQ-OAB, with effect sizes being around 0.3 (Table 4). Even when changes in the ICECAP-A score were assessed based on changes in the frequency of symptoms (Table 5) or based on women’s self-perceived change of symptoms (Table 6), the ICECAP-A was the only measure capturing statistically significant deteriorations in clinical outcomes.

**Table 4:** Responsiveness of the ICECAP-A, EQ-5D-3L, and ICIQ-OAB by self-reported change in symptoms’ bother

	Floor effect (%)		Ceiling effect (%)		Baseline score	Follow-up score	Score change	*P* value	SRM^ǂ^
	Baseline	Follow-up	Baseline	Follow-up	Mean (SD)	Mean (SD)	Mean (SD)		
ICECAP-A									
All women (n = 441)	0.00	0.63	11.72	12.34	0.83 (0.17)	0.81 (0.20)	−0.02 (0.15)	0.02*	−0.11
Increased bother (n = 132)	0.00	0.21	3.14	2.93	0.82 (0.16)	0.78 (0.22)	−0.05 (0.15)	0.00**^,†^	−0.32a
Same bother (n = 46)	0.00	0.00	1.67	0.84	0.78 (0.22)	0.74 (0.22)	−0.03 (0.17)	0.17	−0.21a
Lower bother (n = 263)	0.00	0.42	6.90	8.58	0.84 (0.17)	0.84 (0.19)	0.00 (0.15)	0.86	0.01
EQ-5D-3L									
All women (n = 452)	0.00	0.00	15.90	20.71	0.68 (0.28)	0.66 (0.33)	−0.02 (0.25)	0.15	−0.07
Increased bother (n = 135)	0.00	0.00	5.44	4.81	0.69 (0.28)	0.64 (0.33)	−0.05 (0.24)	0.02*^,†^	−0.21a
Same bother (n = 47)	0.00	0.00	1.67	1.88	0.59 (0.38)	0.57 (0.39)	−0.02 (0.22)	0.50	−0.10
Lower bother (n = 270)	0.00	0.00	8.79	14.02	0.69 (0.26)	0.69 (0.32)	0.00 (0.26)	0.97	0.00
ICIQ-OAB									
All women (n = 454)	0.00	0.00	0.00	1.05	9.20 (2.67)	7.31 (3.33)	−1.89 (3.01)	0.00**^,††^	−0.63b
Increased bother (n = 136)	0.00	0.00	0.00	0.00	8.68 (2.48)	8.76 (2.98)	0.07 (2.16)	0.69	0.03
Same bother (n = 46)	0.00	0.00	0.00	0.00	10.39 (3.42)	10.11(3.52)	−0.28 (2.43)	0.43	−0.12
Lower bother (n = 272)	0.00	0.00	0.00	1.05	9.25 (2.56)	6.11 (2.87)	−3.14 (2.82)	0.00**^,††^	−1.12c

* Significant changes at the 5 % level using a paired *t* test

** Significant changes at the 1 % level using a paired *t* test

^†^Significant changes at the 5 % level using a Wilcoxon rank sum test

^††^Significant changes at the 1 % level using a Wilcoxon rank sum test

^ǂ^The values of 0.2, 0.5 and 0.8 represent the cut-off points for small (a), moderate (b), and large (c) standardised response mean (SRM) effect sizes [32]

**Table 5:** Responsiveness of the ICECAP-A and EQ-5D-3L by change in symptoms’ frequency (i.e. ICIQ-OAB score)

	Baseline score	Follow-up score	Score change	*P* value	SRM^ǂ^
	Mean (SD)	Mean (SD)	Mean (SD)		
ICECAP-A					
Improved (n = 272)	0.85 (0.17)	0.85 (0.18)	0.00 (0.15)	0.96	0.00
Same level (n = 75)	0.79 (0.18)	0.76 (0.21)	−0.03 (0.13)	0.06	−0.23a
Deteriorated (n = 97)	0.80 (0.18)	0.74 (0.22)	−0.06 (0.18)	0.00**^,††^	−0.32a
EQ-5D-3L					
Improved (n = 280)	0.70 (0.26)	0.70 (0.30)	0.00 (0.25)	0.77	0.02
Same level (n = 75)	0.66 (0.31)	0.61 (0.36)	−0.05 (0.24)	0.08^†^	−0.21a
Deteriorated (n = 97)	0.64 (0.31)	0.59 (0.37)	−0.05 (0.26)	0.04*	−0.21a

* Significant changes at the 5 % level using a paired *t* test

** Significant changes at the 1 % level using a paired *t* test

^†^Significant changes at the 5 % level using a Wilcoxon rank sum test

^††^Significant changes at the 1 % level using a Wilcoxon rank sum test

^ǂ^The values of 0.2, 0.5 and 0.8 represent the cut-off points for small (a), moderate (b), and large (c) standardised response mean (SRM) effect sizes [32]

**Table 6:** Responsiveness of the ICECAP-A, EQ-5D-3L, and ICIQ-OAB by self-perceived change of symptoms

	Baseline score	Follow-up score	Score change	*P* value	SRM^ǂ^
	Mean (SD)	Mean (SD)	Mean (SD)		
ICECAP-A					
Improved (n = 136)	0.86 (0.14)	0.86 (0.16)	0.00 (0.14)	0.97	0.00
Same level (n = 104)	0.83 (0.17)	0.81 (0.19)	−0.02 (0.15)	0.11	−0.16
Deteriorated (n = 48)	0.79 (0.19)	0.73 (0.24)	−0.07 (0.15)	0.00**^,†^	−0.45a
EQ-5D-3L					
Improved (n = 140)	0.71 (0.26)	0.71 (0.29)	0.00 (0.21)	0.79	−0.02
Same level (n = 107)	0.64 (0.30)	0.64 (0.33)	0.01 (0.27)	0.84	0.02
Deteriorated (n = 50)	0.65 (0.26)	0.55 (0.39)	−0.10 (0.33)	0.04*	−0.31a
ICIQ-OAB					
Improved (n = 139)	8.90 (2.55)	6.04 (3.06)	−2.86 (3.32)	0.00**^,††^	−0.86c
Same level (n = 113)	9.12 (2.58)	7.94 (3.04)	−1.18 (2.50)	0.00**^,††^	−0.47a
Deteriorated (n = 49)	8.80 (2.70)	8.45 (3.57)	−0.35 (2.63)	0.36	−0.13

* Significant changes at the 5 % level using a paired *t* test

** Significant changes at the 1 % level using a paired *t* test

^†^Significant changes at the 5 % level using a Wilcoxon rank sum test

^††^Significant changes at the 1 % level using a Wilcoxon rank sum test

^ǂ^The values of 0.2, 0.5 and 0.8 represent the cut-off points for small (a), moderate (b), and large (c) standardised response mean (SRM) effect sizes [32]

## Discussion

This paper explored the psychometric properties of the ICECAP-A in relation to the EQ-5D-3L and ICIQ-OAB in a sample of women with lower urinary tract symptoms. This was the first study assessing the construct validity of the ICECAP-A in a clinical group, and the first assessing its responsiveness in a clinical area where symptoms are likely to affect an individual’s quality of life, or well-being, in a much broader sense than conceptualised by conventional health status measures.

The results provided supporting evidence for the acceptability, construct validity, and responsiveness of the ICECAP-A in this context. The ICECAP-A showed high levels of acceptability, with completion rates being above 95 %. In terms of construct validity, a strong correlation was found between the ICECAP-A and EQ-5D-3L index scores and with the EQ-5D-3L attribute of *Anxiety and depression*. Out of the 36 correlations explored between the two measures, only the correlation between the attributes of *Attachment* and *Mobility* was not statistically significant, while from the remaining correlations, 33 (94.3 %) were statistically significant at the 1 % level. Similarly, out of the 22 hypothesised correlations between the ICECAP-A and ICIQ-OAB, 20 (90.9 %) appeared to be statistically significant, with 15 (75 %) of them being significant at the 1 % level.

In terms of discriminative validity, the ICECAP-A was found to have better discriminative properties than EQ-5D-3L and as good as those of the condition-specific questionnaire (ICIQ-OAB), as it was able to detect significant differences in capability-well-being, not only among the BMI categories, and according to the presence or not of detrusor overactivity, but also between the different levels of ‘bother’ from urinary symptoms. In the light of mixed evidence for the association between age and quality of life in this clinical group (see Appendix 2), no significant difference in capability-well-being was hypothesised between age groups. Even though age is expected to inhibit capability and health, this study found no significant differences in terms of health status (EQ-5D-3L) and capability-well-being (ICECAP-A) between those above and below the age of 65. These findings are in line with previous validation work on the ICECAP-A in a general population sample [16] and are potentially attributable to the fact that urinary symptoms might disproportionately affect those employed or more socially engaged, diluting the age effect. The absence of such information did not enable these covariates to be controlled for in the analysis.

The responsiveness analyses explored changes in the ICECAP-A index score in response to changes in the level of ‘bother’ and frequency as well as in response to self-perceived change of urinary symptoms. The results indicated that the ICECAP-A was more responsive to a deterioration of women’s symptoms compared with the EQ-5D-3L in all responsiveness analyses and also compared with the ICIQ-OAB when ‘bother’ and self-perceived change of symptoms were used as anchors. Thus, *deteriorations* in clinical outcomes appeared to be ‘valued’ more highly than *improvements* by the ICECAP-A, in line with previous evidence [17], even though this could be due to the baseline distribution of scores.

The study benefited from a relatively large sample size and the use of longitudinal data, which enabled a thorough assessment of both construct validity and responsiveness. In addition, given that the assumption of normality underpinning parametric tests is often violated in quality of life data, nonparametric tests were also included in the analysis. Although evidence exists in support of parametric tests even in violations of the normality assumption [35], the results obtained from the two tests were sometimes contradictory.

Nevertheless, there are a number of caveats worth highlighting in the interpretation of the study’s findings. First, in the absence of a gold-standard measure of well-being, the psychometric properties of the ICECAP-A could only be investigated against hypothetically developed constructs and imperfect anchors of clinical change. Second, the primary study was designed to test the accuracy and cost-effectiveness of a diagnostic strategy, rather than the clinical effectiveness of an intervention. Because of limitations in the primary data, it is uncertain whether there were other health or well-being impacts, such as an unrelated adverse health event, that a woman might have experienced that could have influenced the generic health or well-being measures of this study. Finally, the primary study targeted only women with symptoms of urinary urgency and frequency, with or without urinary incontinence, and thus, findings are restricted to the specific sample used. Strengths and limitations associated with the primary study, from which the data were drawn, can be found in the full Health Technology Assessment report [23].

There are potentially several reasons explaining the good psychometric performance of the ICECAP-A in this clinical group. First, the ICECAP-A comprises conceptual attributes that capture a broader evaluative space that extends beyond HrQoL to the capability to function in terms of *Stability, Attachment, Autonomy, Achievement* and *Enjoyment*. This allows for more extensive practical and emotional implications from urinary symptoms to be captured. Intuitively, it might be expected that, in this clinical group, symptoms of urgency or incontinence would be significantly correlated with the EQ-5D-3L attribute of *Anxiety and depression* [20, 36, 37]. However, this was not evident in this study. While the EQ-5D-3L attribute of *Usual activities* might capture some broader practical implications of urinary symptoms, the emotional ones appear to be largely missed. This also possibly explains why in this study the EQ-5D-3L was not able to distinguish between different levels of ‘bother’ from urinary symptoms, a finding that confirms previous validation work which found no association between symptom severity and the EQ-5D-3L index score and attributes [38].

Second, the ICECAP-A has more response options than the EQ-5D-3L, which in turn may allow for a greater degree of sensitivity and smaller floor and ceiling effects. In this study, 16 and 21 % of women reported full health at baseline and 6-month follow-up, respectively, whereas approximately 12 % of women reported full capability at the two time-points. Of course, this issue might be ameliorated with the development of the new five-level EQ-5D (EQ-5D-5L) [39]. Finally, another driver of the good performance of the ICECAP-A is the lower statistical dispersion observed in the results, which subsequently made the different statistics more favourable compared to the EQ-5D-3L, even when absolute changes were of similar or smaller magnitude. This might be an implication arising from the wider scale of values generated from the EQ-5D-3L, which can range from −0.594 to 1 and not necessarily between 0 and 1 as the ICECAP-A. This, however, allows for larger changes to be seen, especially when interventions are aimed at those with low levels of health.

More research is required in order to establish the psychometric performance of the ICECAP-A. Comparisons with other capability measures (e.g. ASCOT [40] or OxCap-MH [11]) or other measures of HrQoL (e.g. EQ-5D-5L [39] or SF-6D [41, 42]), and in different settings are required to shed further light on its measurement properties. Given that recent recommendations for the evaluation of social care interventions, published by the National Institute for Health and Care Excellence (NICE) in the UK, suggest a parallel use of an ICECAP measure when capability benefits are relevant [43], further research is required to establish the validity and responsiveness of the ICECAP-A in different social care contexts. Finally, given the limited empirical evidence for the validity and responsiveness of the measure in the evaluation of physical health problems, further research is required to establish the sensitivity of the measure to capture differences and changes in physical health status.

In conclusion, the findings of this study have provided strong evidence for the construct validity and responsiveness of the ICECAP-A and support its use in the economic evaluation of interventions for urinary symptoms in women. Using the ICECAP-A in this context will allow for a more holistic assessment of women’s experience of urinary symptoms and treatment outcomes.

## Appendix 1

Correlations between the ICECAP-A and ICIQ-OAB that were expected by the authors to be significant at the 5 % level (✓) or not (✗) based on available evidence from the literature and their personal opinion before the statistical analysis.

**Table:**

ICIQ-OAB	ICECAP-A					
	Capability index score	Stability	Attachment	Autonomy	Achievement	Enjoyment
ICIQ-OAB score	✓	✓	✗	✓	✓	✓
Frequency of urination (day)	✓	✓	✗	✓	✓	✓
Frequency of urination (night)	✓	✓	✗	✗	✗	✗
Frequency of rush for urination	✓	✓	✗	✓	✓	✓
Frequency of leaking before urination	✓	✓	✗	✓	✓	✓

Correlations were expected to be negative and in the weak range

### Evidence upon which correlations where hypothesised

- Frequency of urination tends to impact on social function, general and mental health and often results in sleep problems [44, 45].
- Urgency and nocturia tend to have a significant impact on quality of life dimensions, such as physical functioning, pain, general health, vitality, social functioning, physical and emotional role, mental health and sleep [44–47].
- Urinary incontinence affects daily life activities, limits behaviour, and also has a psychosocial impact [38, 48].

## Appendix 2

Associations between the ICECAP-A index score and different indicators that were expected by the authors to be significant at the 5 % level (✓) or not (✗) based on available evidence from the literature and their personal opinion before the statistical analysis.

**Table:**

Variables	Expected association	Evidence upon which associations where hypothesised
Age (<65, ≥65)	✗	Evidence for the relationship between age and quality of life among people with symptoms of OAB is contradictory [21, 49, 50]
BMI (normal, overweight, obese)	✓	Evidence for BMI suggests a significant association with quality of life measured with disease-specific and general measures of HrQoL [21, 49–51]
Clinical diagnosis (overactive bladder, mixed incontinence, stress incontinence)	✗	The type of clinical diagnosis among individuals with symptoms of OAB is not a significant determinant of quality of life [49, 52]
Detrusor overactivity (no, yes)	✓	Quality of life appears to be impaired among those with an urodynamically verified detrusor overactivity [53]
Surgery (no, yes)	✗	Evidence for the relationship between quality of life and previous urinary surgery, presence of prolapse or existence of voiding difficulties is scarce and contradictory [20, 45, 50]
Advance prolapse (no, yes)	✗	
Voiding difficulty (no, yes)	✗	
Bother—frequency of urination (day) (≤5, >5)	✓	There is robust evidence, indicating that OAB symptoms severity significantly impacts on quality of life and can be captured by both generic measures, like the EQ-5D, and disease-specific [44, 49, 54, 55]. For the EQ-5D, however, there has been evidence, indicating that severity is not significantly associated with HrQoL [38]
Bother—frequency of urination (night) (≤5, >5)	✓	
Bother—frequency of rush (≤5, >5)	✓	
Bother—frequency of leaking (≤5, >5)	✓	
Total bother of symptoms (low, moderate, high)	✓	

## Footnotes

1 ICEPOP was a UK MRC-funded Health Services Research Collaboration programme on Investigating Choice Experiments for Preferences of Older People; it was the research programme in which the first ICECAP measure was developed.
